# Association between smoking and lack of HIV virological suppression in a cross-sectional study of persons with HIV on antiretroviral therapy in Uganda

**DOI:** 10.1371/journal.pone.0300508

**Published:** 2024-03-20

**Authors:** Adah Tumwegamire, Robin Fatch, Nneka I. Emenyonu, Sara Lodi, Winnie R. Muyindike, Allen Kekibiina, Julian Adong, Christine Ngabirano, Brian Beesiga, Kara Marson, Nakisa Golabi, Moses Kamya, Gabriel Chamie, Judith A. Hahn

**Affiliations:** 1 Global Health Collaborative, Mbarara University of Science and Technology, Mbarara, Uganda; 2 Division of HIV, Infectious Disease and Global Medicine, University of California San Francisco, San Francisco, United States of America; 3 Boston University School of Public Health, Boston, Massachusetts, United States of America; 4 Mbarara Regional Referral Hospital, Mbarara, Uganda; 5 Infectious Diseases Research Collaboration, Kampala, Uganda; 6 Department of Epidemiology and Biostatistics, University of California, San Francisco, CA, United States of America; Makerere University CHS: Makerere University College of Health Sciences, UGANDA

## Abstract

**Background:**

Smoking and alcohol use frequently co-occur and are the leading causes of preventable death in sub-Saharan Africa (SSA) and are common among people living with HIV (PLWH). While alcohol use has been shown to be associated with reduced adherence to antiretroviral treatment (ART), which may affect HIV viral suppression, the independent effect of smoking on HIV outcomes in SSA is unknown. We aimed to 1) describe the prevalence of current smoking and correlates of smoking; 2) assess the association of smoking with viral suppression, adjusting for level of alcohol use; 3) explore the relationship between smoking and CD4 cell count <350 cells/mm^3^, among participants who are virally suppressed.

**Methods:**

We analyzed data from the Drinkers Intervention to Prevent Tuberculosis (DIPT) and the Alcohol Drinkers’ Exposure to Preventive Therapy for TB (ADEPTT) studies conducted in Southwest Uganda. The studies enrolled PLWH who were on ART for at least 6 months and co-infected with latent tuberculosis and dominated with participants who had unhealthy alcohol use. Current smoking (prior 3 months) was assessed by self-report. Alcohol use was assessed using the Alcohol Use Disorders Identification Test-Consumption (AUDIT-C, modified for prior 3 months) and phosphatidylethanol (PEth), an alcohol biomarker. We used logistic regression to estimate the cross-sectional association between smoking and lack of virological suppression (≥40 copies/ml), adjusting for level of alcohol use and other covariates, and to examine the association between smoking and CD4 cell counts among PLWH with viral suppression.

**Results:**

Of the 955 participants enrolled from 2017 to 2021 who had viral load (VL) results, 63% were men, median age was 40 years (interquartile range [IQR] 32–47), 63% engaged in high/very high-risk alcohol use (AUDIT-C≥6 or PEth≥200 ng/mL), and 22% reported smoking in the prior 3 months. Among 865 participants (91%) with viral suppression and available CD4 count, 11% had a CD4 cell count <350 cells/mm^3^. In unadjusted and adjusted analyses, there was no evidence of an association between smoking and lack of virological suppression nor between smoking and CD4 count among those with viral suppression.

**Conclusions:**

The prevalence of smoking was high among a study sample of PLWH in HIV care with latent TB in Southwest Uganda in which the majority of persons engaged in alcohol use. Although there was no evidence of an association between smoking and lack of virological suppression, the co-occurrence of smoking among PLWH who use alcohol underscores the need for targeted and integrated approaches to reduce their co-existence and improve health.

## Introduction

Tobacco smoking and alcohol consumption are major health threats and are attributed factors in more than 8 million and 3.3 million deaths per year, respectively [1,2]. The vast majority (80%) of tobacco-related deaths occur in resource limited countries [3]. In addition, cigarette smoking is more common among individuals with higher alcohol consumption compared to others [4].

Globally, smoking is common among people living with HIV (PLWH), with the prevalence among PLWH being 2–3 times that of the general population [5,6]. While the prevalence of smoking among Ugandan adults (9.6%) is lower compared to global estimates (19.2%) [7], smoking among PLWH in Uganda is twice that of persons not infected with HIV [8].

A recent meta-analysis of 290 hospital-based studies found that smoking was associated with sub-optimal ART adherence and further indicates that structural and socioeconomic conditions, as well as mental health conditions (depression, anxiety) that were associated with smoking are also predictive of suboptimal ART adherence [9]. This raises concern that viral suppression may be impacted by poor adherence among smokers. It has also been suggested that smoking causes immunologic disfunction that could affect viral suppression [10]. However, studies that have examined the association of smoking with viral suppression have reported mixed findings. Several studies have found evidence that smoking is associated with lack of virological suppression in PLWH [11–16], while other studies have shown no association [17] or an inverse association [10]. Since most of these studies were conducted in high income countries [10,12,14,15,17], further research on the association between smoking and lack of virological suppression within Sub-Saharan Africa and low-income countries is needed.

Alcohol use, through its effect on ART non-adherence, is also reported as a risk factor for decreased viral suppression [18]. Because smoking and alcohol use commonly co-occur, studies of the association between smoking and viral suppression need to control for alcohol use. Of the studies that examined whether there was an association between smoking and viral suppression [10–17], three controlled for alcohol use [11,13,17], and of those, only one found a significant association between smoking and viral suppression [13]. To best design interventions to improve HIV outcomes, there is a need to better understand whether smoking impacts HIV viral suppression in patients who receive ART, independent of alcohol use.

Even among those with viral suppression, smoking may contribute to disrupting immune pathways in PLWH [19,20]. However, the literature regarding the association of smoking with CD4 cell count among PLWH on ART has mixed findings. Some studies have linked smoking with poorer CD4 cell count recovery over time after ART initiation [10–12], while other literature found no association [15]. Therefore, there is a need to better understand whether smoking impacts CD4 cell count in PLWH who receive ART.

In this study, we examined the association of smoking on viral suppression among PLWH on ART, controlling for level of alcohol consumption, and examined the association of smoking with CD4 cell count <350 cells/mm^3^ specifically among PLWH who are virally suppressed. Using data from two studies among PLWH and latent tuberculosis (TB), with over-sampling of persons consuming alcohol in Southwest Uganda, we aimed to: 1) describe the prevalence of current smoking and correlates of smoking; 2) assess the association of smoking with viral suppression, adjusting for level of alcohol use; 3) explore the relationship between smoking and CD4 cell count <350 cells/mm^3^, among participants who are virally suppressed.

## Materials and methods

### Study design and setting

We used the baseline data from two longitudinal studies of PLWH with latent TB who were recruited from 2 rural (Ruhoko Health center IV and Rugazi Health center IV) and 2 semi-urban (Mbarara City Council (MCC) and the Mbarara Regional Referral Hospital Immune Suppression syndrome (ISS)) HIV clinics in Southwest Uganda. The Drinkers Intervention to Prevent Tuberculosis (DIPT, NCT03492216) study is a randomized controlled trial (RCT) of economic incentives to promote reduced alcohol consumption and increased adherence to isoniazid (INH). The study incentivized negative urine tests for the short-term alcohol biomarker urine ethyl glucuronide (uEtG), and incentivized INH adherence via positive IsoScreen urine tests, using a factorial design, among 680 people with unhealthy alcohol use (defined below) and co-infected with HIV and latent TB receiving six months of INH [21].The Alcohol Drinkers Exposure to Preventive Therapy for Tuberculosis (ADEPTT, NCT03302299) cohort study is a one-arm trial aimed at examining the safety and tolerability of INH preventive therapy in people co-infected with HIV and TB, including both persons who drink alcohol (n = 200) and persons who abstain from alcohol use (n = 101). These studies are part of the Uganda-Russia-Boston Alcohol Network for Alcohol Research Collaboration on HIV/AIDS (URBAN ARCH) Consortium, previously described [22,23], and collected data using similar study instruments. We used data from the two studies here to reflect a range of alcohol use and urban versus rural settings. We recruited participants from May 2017 through August 2021.

### Study participants and procedures

The screening process to select participants for both studies (DIPT and ADEPTT) consisted of several stages. Eligibility criteria at the initial screening step included individuals who were age≥18 years, living with HIV, fluent in Runyankole (the local language) or English, had been prescribed ART for at least 6 months, lived within 2 hours travel time or 60km of the study site and had no plans to move out of the catchment area, and who had no history of active TB or taking TB preventive medications.

Patients were eligible for the ADEPTT study if they reported current alcohol use (prior 3 months) or abstaining from using alcohol for at least the past year. Patients were eligible for the DIPT study if they recently consumed alcohol, as evidenced by testing positive on a uEtG dipstick test (300 ng/mL cutoff, Confirm Biosciences, San Diego, California), and self-reported unhealthy alcohol use (positive via the Alcohol Use Disorders Identification Test—Consumption (AUDIT-C) (≥3 for women; ≥4 for men), modified to assess for the prior 3 months) [24]. The details of the study participants and procedures in both studies have been published elsewhere [22,23].

### Measurements

We conducted an interviewer-administered structured survey using the Computer Assisted Survey Information Collection (CASIC) system. The participants were interviewed in Runyankole or English depending on the participant’s language of preference. The survey was comprised of questions on demographics, alcohol use, smoking, other substance use, ART medications, general health status, and mental health.

### Specimen collection

Blood samples were collected for HIV viral load (VL) and CD4^+^ cell count and tested at the Mbarara University of Science and Technology (MUST) Clinical Research Laboratory and at Infectious Diseases Research Collaboration (IDRC) Regional Research Laboratory using the BD FACS Presto (TM), PIMA POC CD4 test system (Alere Inc., Waltham, MA) and GeneXpert® Dx System. Dried blood spot (DBS) cards were prepared to test for phosphatidylethanol (PEth), a biomarker of alcohol consumption in the prior 2–3 weeks [25]. DBS were tested for PEth homologue 16:0/18:1 with limit of quantification of 8 ng/mL at the United States Drug Testing Laboratories Inc, Des Plaines, Illinois, USA using previously described methods [26].

### Variables

#### Outcomes

We examined the following outcomes: non-suppressed HIV, defined as HIV RNA levels more than 40 copies/ml and low CD4, defined as CD4 count <350 cells/mm^3^.

#### Independent variables

*Current smoking*. We ascertained current smoking status during the structured interview by asking participants whether they had smoked in the past 3 months (yes/no). Participants reporting current smoking were also asked to report the number of smoking days in the prior 30 days.

*Alcohol use*. We measured alcohol use by self-report using the Alcohol Use Disorders Identification Test—Consumption (AUDIT-C) [24], modified to represent past 3 months alcohol use. We used standard AUDIT-C cutoffs for medium risk alcohol use (≥3 for women or ≥4 for men) [24], and ≥6 for high/very high risk drinking [27]. We used a cutoff of PEth ≥ 50 ng/ml as an indicator of unhealthy drinking, as in prior research [28], and a cutoff of PEth ≥ 200 ng/ml to indicate excessive drinking [29]. We used a combination of AUDIT-C and PEth to define the level of alcohol use, as follows: “abstaining/low-risk alcohol use”: AUDIT-C negative and PEth < 50 ng/ml; “medium risk”: AUDIT-C ≥3 for women or ≥4 for men but < 6 or PEth between 50 and 199 ng/ml; “high/very high risk”: AUDIT-C ≥ 6 or PEth ≥ 200 ng/ml. Persons with past alcohol use were defined as participants who reported no past year alcohol use (AUDIT = 0) and who had any past history of alcohol use.

*Confounders*. We collected participant characteristics that included age, gender, level of education, general physical health, marital status, religiosity, adherence to ART, and social desirability on the study questionnaire. We measured social desirability using the Marlow-Crowne 28-item scale (possible range 0–28) [30]. We measured spirituality/religiosity using the Duke University Religion Index (DUREL Scale) [31] and used the intrinsic religiosity subscale of the DUREL to describe the participants’ religious beliefs and experience in religious matters. The scores on these scales were treated as continuous variables. We measured self-reported ART adherence in prior 30 days with response options of very poor, poor, fair, good, very good and excellent [32], dichotomized as excellent/very good versus good/fair/poor/very poor. We measured symptoms of depression using the Center for Epidemiologic Studies Depression (CESD) Scale, which has 20 questions, each scaled from 0 to 3, with a positive assessment indicating risk for depression if the score was ≥16 [33,34]. We assessed general health status using the first question of the Medical Outcomes Study-HIV (MOS-HIV) Health Survey that asked: In general, would you say your health is excellent, very good, good, fair, or poor? The MOS-HIV has been used before as a validated measure of health in Uganda [35].

#### Statistical analysis

We included 955 individuals who enrolled in the parent studies who had viral load results. Sample characteristics were described using proportions for variables that were categorical and medians and inter-quartile range (IQR) for continuous variables. We examined Spearman correlations between variables for assessment of collinearity of the independent variables we planned to include in the multivariable models. We found that alcohol use and study site were correlated (Spearman rank correlation>0.40), thus we fit random effects models with study site as a random effect to account for clustering by site for the multivariable analyses.

We examined the association between participants’ characteristics and current smoking using Chi-squared tests for categorical variables and Mann-Whitney tests for continuous variables. We used random effects models with a logit link to examine the relationships between current smoking with lack of virological suppression and between current smoking and low CD4 count among participants who were virally suppressed. All models were adjusted for level of alcohol use, for gender and age as these covariates have been associated with viral suppression and/or CD4 count in the literature [36,37] and for social desirability scale because of prior research showing under-report of potentially stigmatized behaviors [38,39].

We also conducted three exploratory analyses for each outcome. The first exploratory model was additionally adjusted for self-reported ART adherence, to explore the potential mediating effect of adherence. The second exploratory model included an interaction term between smoking and alcohol use, to explore the potential synergistic effect of unhealthy alcohol use and smoking. We conducted additional random effect regression models as part of a third exploratory analysis to explore the effect of the number of days smoked by including the number of days smoked rather than smoking in the prior 3 months (yes/no). We conducted additional sensitivity analyses to explore whether there was an effect on our results due to the inclusion of those who previously drank alcohol but who may have quit due to health issues (“sick quitting”) [40], by excluding participants who had not consumed alcohol in the prior year, but who had reported any past history of alcohol use. Finally, we conducted additional sensitivity analyses to assess whether results may be different by ART type; we conducted multivariable models, adjusted for ART regimen type (NNRTI-based, INSTI-base, PI-based).

#### Ethical considerations

Participants provided written informed consent prior to enrollment into the studies. All protocols were approved by the institutional review boards of the University of California, San Francisco (UCSF) and Boston University, the Mbarara University of Science and Technology Research Ethics Committee (MUST-REC), the Makerere University School of Medicine Research Ethics Committee (SOMREC), and the Ugandan National Council for Science and Technology (UNCST). In the study consent, the participants gave permission to be contacted after the study ended.

## Results

The DIPT (n = 680) and ADEPTT (n = 301) studies enrolled participants from May 2017 through August 2021. Seventeen of the 680 persons enrolled in the DIPT study did not have viral load results leaving 663 for analysis; nine of 301 participants from the ADEPTT study were missing viral load results leaving 292 for analysis, for a total of 955. The median age was 40 years (interquartile range [IQR]: 32–47 “Table 1”. The majority of participants were men (63.4%), married (61.0%), had at most a primary level of education (77.7%), and reported very good to excellent general health (51.4%). Twenty-two percent (22.2%) reported smoking tobacco in the prior 3 months. For alcohol use, 17.0% were in the abstaining/low risk drinking group, 20.4% were in the medium risk group, and 62.6% were in the high/very high-risk group. Eighty-eight participants (9.2%) had lack of virological suppression and the median log10 viral load was 2.3 copies/ml^3^ (IQR: 1.8–3.7). Among participants with viral suppression (n = 867) whose CD4 counts were available (n = 865), 10.6% had a CD4 cell count <350 cells/ml^3^ and the median CD4 count in this group was 283 (IQR: 225–324).

**Table 1:** Participant characteristics and bivariate associations with smoking (n = 955).

		Current smoking (past 3 months)		
	N (%) or median [IQR]	No (n = 743) N (%)	Yes (n = 212) N (%)	X2 or Mann-Whitney z (p-value)
Age	40 [32–47]	40 [32–46]	40 [34–48]	-1.67 (0.095)
Gender				61.92 (<0.001)
Women	350 (36.7)	321 (91.7)	29 (8.3)	
Men	605 (63.4)	422 (69.8)	183 (30.3)	
Social desirability score	20 [17–22]	20 [18–22]	19 [16.6–22]	2.46 (0.014)
ART adherence				0.37 (0.541)
Excellent/Very good	762 (79.8)	596 (78.2)	166 (21.8)	
Good/Fair/Poor/Very poor	193 (20.2)	147 (76.2)	46 (23.8)	
Any lifetime smoking				
No	637 (66.7)	637 (100.0)	0 (0.0)	
Yes	318 (33.3)	106 (33.3)	212 (66.7)	
Current smoking (prior 3 months)				
No	743 (77.8)	743 (100.0)	0 (0.0)	
Yes	212 (22.2)	0 (0.0)	212 (100.0)	
Alcohol use*				52.53 (<0.001)
Abstainers/low-risk	161 (17.0)	153 (95.0)	8 (5.0)	
Medium-risk	194 (20.4)	166 (85.6)	28 (14.4)	
High/very high-risk	594 (62.6)	419 (70.5)	175 (29.5)	
Persons with past alcohol use**				9.11 (0.003)
No	896 (94.3)	689 (76.9)	207 (23.1)	
Yes	54 (5.7)	51 (94.4)	3 (5.6)	
Detectable viral load				0.47 (0.495)
No	867 (90.8)	672 (77.5)	195 (22.5)	
Yes	88 (9.2)	71 (80.7)	17 (19.3)	
CD4 cell count <350, among those virally suppressed (n = 865)				0.19 (0.666)
No	773 (89.4)	598 (77.4)	175 (22.6)	
Yes	92 (10.6)	73 (79.4)	19 (20.7)	
Study site				14.04 (0.003)
ISS	378 (39.6)	317 (83.9)	61 (16.1)	
MMC	245 (25.7)	180 (73.5)	65 (26.5)	
Ruhoko	108 (11.3)	83 (76.9)	25 (23.2)	
Rugazi	224 (23.5)	163 (72.8)	61 (27.2)	

Alcohol use defined as: “Abstainers/low-risk” AUDIT-C negative and PEth < 50; “medium-risk” AUDIT-C positive but AUDIT-C < 6 and/or 50 < = PEth < 200; “high/very high-risk” AUDIT-C > = 6 or PEth > = 200.

** Persons with past alcohol use were defined as participants who were past year AUDIT = 0 who had any past history of alcohol use.

### Smoking

Among the 212 participants who reported smoking in the prior 3 months, the median number of days smoked in the prior month was 30 days (IQR: 30–30). Thirty percent (30.3%) of men reported current smoking compared to 8.3% of women. The level of alcohol use was associated with current smoking “Table 1”; 5.0% of participants in the alcohol abstinence/low-risk drinking group reported current smoking, compared to 14.4% of those in the medium-risk drinking group and 29.5% of those in the high/very high-risk drinking group. General health status (worse), social desirability score (lower) and marital status (not married) were also significantly associated with current smoking. There were no significant differences in reporting current smoking by depressive symptoms, intrinsic religiosity, or ART adherence.

### Lack of virological suppression

In unadjusted analyses, there was no evidence of an association between current smoking or level of alcohol use with viral non-suppression “Table 2”. 948 participants were included in the multivariable model examining lack of virological suppression. Seven of 955 participants were excluded due to missing data; 6 were missing alcohol use and 1 was missing social desirability score. The adjusted odds ratio (aOR) for lack of virological suppression for persons who currently smoke compared to those who do not currently smoke was 0.74 (95% confidence interval (CI): 0.42–1.32) "Table 3". There was no statistical evidence of an association between alcohol use and viral suppression. Age was significantly associated with lack of virological suppression, with lower odds of non-suppression with increasing age (aOR 0.96, 95% CI: 0.94–0.99, p = 0.004). In the first exploratory model which additionally adjusted for ART adherence, there was evidence of an association between ART adherence and lack of virological suppression with those reporting excellent or very good ART adherence having lower odds of lack of virological suppression compared to those reporting good/fair/poor/very poor adherence (aOR 0.55, 95% CI: 0.34–0.90, p = 0.018), but the aOR for the relationship between smoking and lack of virological suppression in this model again showed no evidence of associations with viral suppression. The second exploratory model including an interaction term between smoking and level of alcohol use did not converge, most likely due to the high overlap between smoking and alcohol use, and therefore no results are shown. We also conducted a sensitivity analysis by running the multivariable model for lack of virological suppression including number of days smoked rather than current smoking, and the results were very similar to the model including current smoking (data not shown).

**Table 2:** Unadjusted odds ratios of lack of virological suppression (n = 955).

	Lack of virological suppression			
	No (n = 867) N (row %)	Yes (n = 88) N (row %)	Unadjusted Odds Ratio (95% CI)	p-value
Current smoking				0.496
No	672 (90.4)	71 (9.6)	1.00	
Yes	195 (92.0)	17 (8.0)	0.83 (0.47, 1.43)	
Alcohol use*				0.393
Abstainers/low-risk	149 (92.6)	12 (7.5)	1.00	
Medium-risk	179 (92.3)	15 (7.7)	1.04 (0.47, 2.29)	
High/very high-risk	533 (89.7)	61 (10.3)	1.42 (0.75, 2.71)	
Age (median [IQR])	40 [33–47]	35 [30–43.5]	0.97 (0.94–0.99)	0.007
Gender				
Women	322 (92.0)	28 (8.0)	1.00	
Men	545 (90.1)	60 (9.9)	1.27 (0.79, 2.02)	0.324
Social desirability score (median [IQR])	20 [17–22]	20 [17–22]	1.01 (0.95, 1.07)	0.766
ART adherence				0.011
Good/Fair/Poor/Very poor	166 (86.0)	27 (14.0)	1.00	
Excellent/Very good	701 (92.0)	61 (8.0)	0.54 (0.33, 0.87)	
Study site				0.973
ISS	344 (90.2)	34 (9.0)	1.00	
MMC	221 (90.2)	24 (9.8)	1.10 (0.63, 1.90)	
Ruhoko	99 (91.7)	9 (9.4)	0.92 (0.43, 1.98)	
Rugazi	203 (90.6)	21 (9.4)	1.05 (0.59, 1.85)	

* Alcohol use defined as: “Abstainers/low-risk” AUDIT-C negative and PEth < 50; “medium-risk” AUDIT-C positive but AUDIT-C < 6 and/or 50 < = PEth < 200; “high/very high-risk” AUDIT-C > = 6 or PEth > = 200.

**Table 3:** Adjusted odds ratios (aOR) and 95% confidence intervals (CI) from multivariable mixed effect logistic regression models of lack of virological suppression (n = 948).

	aOR (95% CI)*	p-value	aOR (95% CI)	p-value
Current smoking		0.314		0.318
No	1.00		1.00	
Yes	0.74 (0.42, 1.32)		0.74 (0.42, 1.33)	
Alcohol use**		0.533		0.622
Abstainers/low-risk	1.00		1.00	
Medium-risk	0.95 (0.42, 2.11)		0.92 (0.41, 2.05)	
High/very high-risk	1.29 (0.65, 2.57)		1.22 (0.61, 2.44)	
Age	0.96 (0.94, 0.99)	0.003	0.96 (0.94, 0.99)	0.004
Gender		0.170		0.135
Women	1.00		1.00	
Men	1.44 (0.85, 2.43)		1.49 (0.88, 2.52)	
Social desirability score	1.03 (0.96, 1.10)	0.401	1.03 (0.97, 1.10)	0.324
ART adherence				0.018
Good/Fair/Poor/Very poor	-		1.00	
Excellent/Very good	-		0.55 (0.34, 0.90)	

*models using meqrlogit in Stata (QR decomposition), with random effects for site.

** Alcohol use defined as: “Abstainers/low-risk” AUDIT-C negative and PEth < 50; “medium-risk” AUDIT-C positive but AUDIT-C < 6 and/or 50 < = PEth < 200; “high/very high-risk” AUDIT-C > = 6 or PEth > = 200.

### Low CD4 count among those with viral suppression

In unadjusted analyses among the 865 participants with viral suppression, there was no evidence of an association between current smoking and low CD4 (OR 0.89; 95% CI: 0.52–1.51),”Table 4”. The aOR for low CD4 for persons who currently smoke was 0.64 (95% CI: 0.37–1.11), compared to those who do not currently smoke “Table 5”. There was also no evidence of an association between level of alcohol use and low CD4. The adjusted odds of low CD4 were higher for men compared to women (aOR 3.61; 95% CI: 1.89–6.90). In the third model that additionally adjusted for ART adherence, there was no evidence of an association between ART adherence and low CD4 among PLWH with viral suppression. The second model including an interaction term between smoking and level of alcohol use did not converge (data not shown). We also ran the model for low CD4 including number of days smoked and the results were very similar to the model that included current smoking (data not shown).

**Table 4:** Unadjusted odds ratios of CD4 < 350, study participants with viral suppression (n = 865).

	CD4 < 350			
	No (n = 773) N (row %)	Yes (n = 92) N (row %)	Unadjusted Odds Ratio (95% CI)	p-value
Age (median [IQR])	40 [33–46]	43 [32–50]	1.03 (1.01, 1.05)	0.011
Gender				<0.001
Women	307 (95.6)	14 (4.4)	1.00	
Men	466 (85.7)	78 (14.3)	3.67 (2.04, 6.60)	
Social desirability score (median [IQR])	20 [17–22]	20 [17.4–23]	1.02 (0.96, 1.08)	0.535
ART adherence				0.493
Good/Fair/Poor/Very poor	149 (90.9)	15 (9.2)	1.00	
Excellent/Very good	624 (89.0)	77 (11.0)	1.23 (0.69, 2.19)	
Current smoking				0.666
No	598 (89.1)	73 (10.9)	1.00	
Yes	175 (90.2)	19 (9.8)	0.89 (0.52, 1.51)	
Alcohol use*				0.040
Abstainers/low-risk	141 (94.6)	8 (5.4)	1.00	
Medium-risk	163 (91.1)	16 (8.9)	1.73 (0.72, 4.16)	
High/very high-risk	465 (87.6)	66 (12.4)	2.50 (1.17, 5.34)	
Study site				0.006
ISS	322 (93.6)	22 (6.4)	1.00	
MMC	195 (89.0)	24 (11.0)	1.80 (0.98, 3.30)	
Ruhoko	84 (84.9)	15 (15.2)	2.61 (1.30, 5.26)	
Rugazi	172 (84.7)	31 (15.3)	2.64 (1.48, 4.70)	

* Alcohol use defined as: “Abstainers/low-risk” AUDIT-C negative and PEth < 50; “medium-risk” AUDIT-C positive but AUDIT-C < 6 and/or 50 < = PEth < 200; “high/very high-risk” AUDIT-C > = 6 or PEth > = 200.

**Table 5:** Adjusted Odds Ratios (aOR) and 95% Confidence Intervals (CI) from multivariable mixed effect logistic regression models* of CD4 < 350, study participants with viral suppression (n = 858).

	aOR (95% CI)	p-value	aOR (95% CI)	p-value
Current smoking		0.115		0.123
No	1.00		1.00	
Yes	0.64 (0.37, 1.11)		0.65 (0.37, 1.13)	
Alcohol use**		0.648		0.630
Abstainers/low-risk	1.00		1.00	
Medium-risk	1.31 (0.52, 3.34)		1.31 (0.52, 3.35)	
High/very high-risk	1.49 (0.63, 3.54)		1.51 (0.63, 3.60)	
Age	1.02 (1.00, 1.04)	0.079	1.02 (1.00, 1.05)	0.077
Gender		<0.001		<0.001
Female	1.00		1.00	
Male	3.61 (1.89, 6.90)		3.57 (1.86, 6.83)	
Social desirability score	1.02 (0.96, 1.09)	0.556	1.02 (0.95, 1.09)	0.575
ART adherence				0.406
Good/Fair/Poor/Very poor	-		1.00	
Excellent/Very good	-		1.30 (0.70, 2.40)	

*models using meqrlogit in Stata (QR decomposition), with random effects for site.

** Alcohol use defined as: “Abstainers/low-risk” AUDIT-C negative and PEth < 50; “medium-risk” AUDIT-C positive but AUDIT-C < 6 and/or 50 < = PEth < 200; “high/very high-risk” AUDIT-C > = 6 or PEth > = 200.

### Sensitivity analyses

We conducted sensitivity analyses by running multivariable models for lack of virological suppression 1) excluding 54 persons with past alcohol use, and 2) additionally adjusted for ART type. The results were similar (data not shown) to our other findings; i.e., there was no evidence of an independent association of current smoking or level of alcohol use with lack of virological suppression. In similar analyses of low CD4, there was no evidence of an independent association of current smoking or level of alcohol use with low CD4 among those with viral suppression, after excluding those with past alcohol use or adjusting for ART regimen type.

## Discussion

In this study of PLWH with latent TB and dominated for alcohol use, there was a high proportion engaging in smoking (22.2%), which is higher than reported in the general population in Uganda (7.9%) [41]. Smoking was more common among men compared to women and among those with high-risk alcohol use. We found no association between smoking and lack of virological suppression; however, viral suppression was high (90.8%). Our findings were similar to another study of two longitudinal cohorts of PLWH with alcohol use in Boston, USA [17]; however, they are in contrast with findings of studies conducted in the USA [14,15] and Cameroon [16], that reported associations between smoking and lack of HIV virological suppression. An explanation for the lack of consistent findings may be that the level of non-adherence needed to result in lack of HIV virological suppression may be quite high, especially with the fairly recent advent and broad uptake of more forgiving second generation integrase strand transfer inhibitor (INSTI)-based regimens [42–44]. As expected, poor ART adherence was associated with lack of virological suppression, and younger age was also associated with lack of virological suppression [45–47].

The proportion with low CD4 count was also low in this population (10.6% had CD4 count<350 copies/mm^3^ among those with viral suppression), and we observed no association between smoking and low CD4 count among those with viral suppression. These findings are similar to another study from two longitudinal cohorts of PLWH who consume alcohol [17] but contrast with two longitudinal cohort studies done in the USA [12,48]. Mixed findings may be partly due to the use of different smoking measures.

This study has several limitations. First, information on smoking was self-reported, and smoking may be under-reported due to social desirability bias [49,50] and recall bias [51,52]. We did not include a biomarkers for smoking to augment self-report, since it was not the focus of these studies. Regardless of these limitations, our study enrollment of a large and multi-site population of PLWH who engage in biomarker-measured alcohol use is a strength. A strength was that we used an objective biomarker to measure alcohol use.

In summary, while smoking was common, especially among the men, we did not find significant associations between smoking and either lack of virological suppression or low CD4, unadjusted or adjusted for alcohol use. This analysis reveals a substantial incidence of tobacco smoking among PLWH who consume alcohol in Southwest Uganda, which constitutes a significant health risk. However, the study indicates that tobacco smoking might not have a direct correlation with viral suppression or CD4 count in PLWH.

## Supporting information

S1 Checklist Strengthening The Reporting of Observational Studies in Epidemiology (STROBE) checklist. (DOCX)

S1 Data (XLSX)
