# Supplementary material for: Association between smoking and lack of HIV virological suppression in a cross-sectional study of persons with HIV on antiretroviral therapy in Uganda
Source: PLoS One. 2024 Mar 20;19(3):e0300508. doi: 10.1371/journal.pone.0300508 (PMC10954112; doi:10.1371/journal.pone.0300508)
Supplement: S1 Checklist — (DOCX) [file pone.0300508.s001.docx]

STROBE Statement—checklist of items that should be included in reports of observational studies

|  | Item No. | Recommendation | Page  No. | Relevant text from manuscript |
| --- | --- | --- | --- | --- |
| **Title and abstract** | 1 | 1. Indicate the study’s design with a commonly used term in the title or the abstract | 1 | Association between smoking and lack of HIV virological suppression among persons with HIV who engage in alcohol use on antiretroviral therapy in Uganda. |
|  |  | (*b*) Provide in the abstract an informative and balanced summary of what was done and what was found | 3-4 | We analysed data from the Drinkers Intervention to Prevent Tuberculosis (DIPT) and the Alcohol Drinkers’ Exposure to Preventive Therapy for TB (ADEPTT) studies conducted in Southwest Uganda. Current smoking (prior 3 months) was assessed by self-report. Alcohol use was assessed using the Alcohol Use Disorders Identification Test-Consumption (AUDIT-C, modified for prior 3 months) and phosphatidylethanol (PEth), an alcohol biomarker. We used logistic regression to estimate the association between smoking and lack of virological suppression (≥40 copies/ml), adjusting for level of alcohol use and other covariates, and to examine the association between smoking and CD4 cell counts among PLWH with viral suppression.  In unadjusted and adjusted analyses, there was no evidence of an association between smoking and lack of virological suppression nor between smoking and CD4 count among those with viral suppression.  The prevalence of smoking was high among a study sample of PLWH in HIV care with latent TB in Southwest Uganda in which the majority of persons engaged in alcohol use. |
| Introduction | | | |  |
| Background/rationale | 2 | Explain the scientific background and rationale for the investigation being reported | 5-7 | Tobacco smoking and alcohol consumption are major health threats and are attributed factors in more than 8 million and 3.3 million deaths per year, respectively.  Globally, smoking is common among people living with HIV (PLWH), with the prevalence among PLWH being 2-3 times that of the general population. While the prevalence of smoking among Ugandan adults (9.6%) is lower compared to global estimates (19.2%), smoking among PLWH in Uganda is twice that of persons not infected with HIV.  Alcohol use, through its effect on ART non-adherence, is also reported as a risk factor for decreased viral suppression.  Because smoking and alcohol use commonly co-occur, studies of the association between smoking and viral suppression need to control for alcohol use.  Even among those with viral suppression, smoking and alcohol use may both be contributory factors to disrupting immune pathways in PLWH. However, the literature regarding the association of smoking with CD4 cell count among PLWH on ART has mixed findings. Some studies have linked smoking with poorer CD4 cell count recovery over time after ART initiation. Other literature found no association. Therefore, there is a need to better understand whether smoking and alcohol use independently impact CD4 cell count in PLWH who receive ART. |
| Objectives | 3 | State specific objectives, including any prespecified hypotheses | 7 | 1) Describe the prevalence of current smoking and correlates of smoking.  2) Assess the association of smoking with viral suppression, adjusting for level of alcohol use.  3) Explore the relationship between smoking and CD4 cell count <350 cells/mm^3^, among participants who are virally suppressed. |
| Methods | | | |  |
| Study design | 4 | Present key elements of study design early in the paper | 7-8 | The Drinkers Intervention to Prevent Tuberculosis (DIPT) study is a randomized controlled trial (RCT) of economic incentives to promote reduced alcohol consumption and increased adherence to isoniazid (INH).The Alcohol Drinkers Exposure to Preventive Therapy for Tuberculosis (ADEPTT) study is aimed at examining the safety and tolerability of INH preventive therapy in people co-infected with HIV and TB, including both persons who drink alcohol and persons who abstain from alcohol use. |
| Setting | 5 | Describe the setting, locations, and relevant dates, including periods of recruitment, exposure, follow-up, and data collection | 7-9 | This analysis was conducted using the baseline data from two studies of PLWH with latent TB who were recruited from 2 rural (Ruhoko Health center IV and Rugazi Health center IV) and 2 semi-urban (Mbarara City Council (MCC) and the Mbarara Regional Referral Hospital Immune Suppression syndrome (ISS)) HIV clinics in Southwest Uganda. We recruited participants from May 2017 through August 2021. The DIPT study incentivized negative urine tests for the short-term alcohol biomarker urine ethyl glucuronide (uEtG), and incentivized INH adherence via positive IsoScreen urine tests, using a factorial design, among 680 people with unhealthy alcohol use (defined below) and co-infected with HIV and latent TB receiving six months of INH. The ADEPTT study is aimed at examining the safety and tolerability of INH preventive therapy in people co-infected with HIV and TB, including both persons who drink alcohol (n=200) and persons who abstain from alcohol use (n=101). |
| Participants | 6 | (*a*) *Cohort study*—Give the eligibility criteria, and the sources and methods of selection of participants. Describe methods of follow-up  *Case-control study*—Give the eligibility criteria, and the sources and methods of case ascertainment and control selection. Give the rationale for the choice of cases and controls  *Cross-sectional study*—Give the eligibility criteria, and the sources and methods of selection of participants | 8-9 | The screening process to select participants for both studies (DIPT and ADEPTT) consisted of several stages. Eligibility criteria at the initial screening step included individuals who were age≥18 years, living with HIV, fluent in Runyankole (the local language) or English, had been prescribed ART for at least 6 months, lived within 2 hours travel time or 60km of the study site and had no plans to move out of the catchment area, and who had no history of active TB or taking TB preventive medications.  Patients were eligible for the ADEPTT study if they reported current alcohol use (prior 3 months) or abstaining from using alcohol for at least the past year.  Patients were eligible for the DIPT study if they recently consumed alcohol, as evidenced by testing positive on a uEtG dipstick test , and self-reported unhealthy alcohol use (positive via the Alcohol Use Disorders Identification Test - Consumption (AUDIT-C) (≥3 for women; ≥4 for men), modified to assess for the prior 3 months). |
|  |  | (*b*) *Cohort study*—For matched studies, give matching criteria and number of exposed and unexposed  *Case-control study*—For matched studies, give matching criteria and the number of controls per case |  |  |
| Variables | 7 | Clearly define all outcomes, exposures, predictors, potential confounders, and effect modifiers. Give diagnostic criteria, if applicable | 10-11 | Outcomes: non-suppressed HIV, defined as HIV RNA levels more than 40 copies/ml at study screening and low CD4, defined as CD4 count <350 cells/mm3 at study screening.  Independent variables  Current smoking: We ascertained current smoking during the structured interview by asking participants whether they had smoked in the past 3 months, and the number of smoking days in the prior 30 days among participants with current smoking.  Alcohol use: Alcohol use defined as: “abstainers/low-risk” AUDIT-C negative and PEth < 50; “medium-risk” AUDIT-C positive but AUDIT-C < 6 and/or 50 <= PEth < 200; “high/very high-risk” AUDIT-C >= 6 or PEth >= 200.  Confounders: We collected participant characteristics that included age, gender, level of education, general physical health, marital status, religiosity, adherence to ART, and social desirability on the study questionnaire. |
| Data sources/ measurement | 8* | For each variable of interest, give sources of data and details of methods of assessment (measurement). Describe comparability of assessment methods if there is more than one group | 9-11 | Outcomes: Blood samples were collected for baseline HIV viral load (VL) and CD4+ cell count and tested at the Mbarara University of Science and Technology (MUST) Clinical Research Laboratory and at Infectious Diseases Research Collaboration (IDRC) Regional Research Laboratory using the BD FACS Presto (TM), PIMA POC CD4 test system (Alere Inc., Waltham, MA) and GeneXpert® Dx System.  Current smoking: We ascertained current smoking during the structured interview by asking participants whether they had smoked in the past 3 months, and the number of smoking days in the prior 30 days among participants with current smoking.  Alcohol use: We measured alcohol use by self-report using the Alcohol Use Disorders Identification Test - Consumption (AUDIT-C), modified to represent the past 3 months alcohol use, augmented by PEth. We used standard AUDIT-C cut-offs for medium risk alcohol use (≥3 for women or ≥4 for men), and ≥6 for high/very high-risk drinking. We used PEth ≥ 50 ng/ml as an indicator of unhealthy drinking, as in prior research, and a cut-offs PEth ≥ 200 ng/ml to indicate excessive drinking. We used a combination of AUDIT-C and PEth to define the level of alcohol use, as follows: “abstaining/low-risk alcohol use”: AUDIT-C negative and PEth < 50 ng/ml; “medium risk”: AUDIT-C (≥3 for women or ≥4 for men but < 6 or PEth between 50 and 199 ng/ml; “high/very high risk”: AUDIT-C ≥ 6 or PEth ≥ 200 ng/ml. Persons with past alcohol use were defined as participants who reported no past year alcohol use (AUDIT scores of zero) and any past history of alcohol use.  Confounders  We collected participant characteristics that included age, gender, level of education, general physical health, marital status, religiosity, adherence to ART, and social desirability on the study questionnaire. We measured social desirability using the Marlow-Crowne 28-item scale (possible range 0-28). We measured spirituality/religiosity using the Duke University Religion Index (DUREL Scale) and used the intrinsic religiosity subscale of the DUREL to describe the participants’ religious beliefs and experience in religious matters. The scores on these scales were treated as continuous variables. We measured self-reported ART adherence in prior 30 days with response options of very poor, poor, fair, good, very good and excellent, dichotomized as excellent/very good versus good/fair/poor/very poor. We measured symptoms of depression using the Center for Epidemiologic Studies Depression (CESD) Scale, which has 20 questions, each scaled from 0 to 3, with a positive assessment indicating risk for depression if the score was ≥16. |
| Bias | 9 | Describe any efforts to address potential sources of bias | 12 | All models were adjusted for level of alcohol use, for gender and age as these covariates have been associated with viral suppression and/or CD4 count in the literature and for social desirability scale because of prior research showing under-report of potentially stigmatized behaviours.  We also conducted three exploratory analyses for each outcome. The first exploratory model was additionally adjusted for self-reported ART adherence, to explore the potential mediating effect of adherence. The second exploratory model included an interaction term between smoking and alcohol use, to explore the potential synergistic effect of unhealthy alcohol use and smoking. We conducted additional random effect regression models as part of a third exploratory analysis to explore the effect of the number of days smoked by including the number of days smoked rather than smoking in the prior 3 months (yes/no). |
| Study size | 10 | Explain how the study size was arrived at |  |  |

Continued on next page

| Quantitative variables | 11 | Explain how quantitative variables were handled in the analyses. If applicable, describe which groupings were chosen and why | 12-13 | Sample characteristics were described using proportions for variables that were categorical and medians and inter-quartile range (IQR) for continuous variables. We examined Spearman correlations between variables for assessment of collinearity of the independent variables we planned to include in the multivariable models. We found that alcohol use and study site were correlated (Spearman rank correlation>0.40), thus we fit random effects models with study site as a random effect to account for clustering by site for the multivariable analyses.  We examined the association between participants’ baseline characteristics and current smoking using Chi-squared tests for categorical variables and Mann-Whitney tests for continuous variables. We used random effects models with a logit link to examine the relationships between current smoking with lack of virological suppression and between current smoking and low CD4 count among participants who were virally suppressed. |
| --- | --- | --- | --- | --- |
| Statistical methods | 12 | (*a*) Describe all statistical methods, including those used to control for confounding | 12-13 | Sample characteristics were described using proportions for variables that were categorical and medians and inter-quartile range (IQR) for continuous variables. We examined Spearman correlations between variables for assessment of collinearity of the independent variables we planned to include in the multivariable models. We found that alcohol use and study site were correlated (Spearman rank correlation>0.40), thus we fit random effects models with study site as a random effect to account for clustering by site for the multivariable analyses.  We examined the association between participants’ baseline characteristics and current smoking using Chi-squared tests for categorical variables and Mann-Whitney tests for continuous variables. We used random effects models with a logit link to examine the relationships between current smoking with lack of virological suppression and between current smoking and low CD4 count among participants who were virally suppressed. All models were adjusted for level of alcohol use, for gender and age as these covariates have been associated with viral suppression and/or CD4 count in the literature and for social desirability scale because of prior research showing under-report of potentially stigmatized behaviours.  We also conducted three exploratory analyses for each outcome. The first exploratory model was additionally adjusted for self-reported ART adherence, to explore the potential mediating effect of adherence. The second exploratory model included an interaction term between smoking and alcohol use, to explore the potential synergistic effect of unhealthy alcohol use and smoking. We conducted additional random effect regression models as part of a third exploratory analysis to explore the effect of the number of days smoked by including the number of days smoked rather than smoking in the prior 3 months (yes/no). We conducted additional sensitivity analyses to explore whether there was an effect on our results due to the inclusion of those who previously drank alcohol but who may have quit due to health issues (“sick quitting”), by excluding participants who had not consumed alcohol in the prior year, but who had reported any past history of alcohol use. Finally, we conducted additional sensitivity analyses to assess whether results may be different by ART type; we conducted multivariable models, adjusted for ART regimen type (NNRTI-based, INSTI-base, PI-based). |
|  |  | (*b*) Describe any methods used to examine subgroups and interactions | 12 | We conducted three exploratory analyses for each outcome. The first exploratory model was additionally adjusted for self-reported ART adherence, to explore the potential mediating effect of adherence. The second exploratory model included an interaction term between smoking and alcohol use, to explore the potential synergistic effect of unhealthy alcohol use and smoking. We conducted additional random effect regression models as part of a third exploratory analysis to explore the effect of the number of days smoked by including the number of days smoked rather than smoking in the prior 3 months (yes/no). |
|  |  | (*c*) Explain how missing data were addressed | 12 | We included all individuals who enrolled in the parent studies who had viral load results |
|  |  | (*d*) *Cohort study*—If applicable, explain how loss to follow-up was addressed  *Case-control study*—If applicable, explain how matching of cases and controls was addressed.  *Cross-sectional study*—If applicable, describe analytical methods taking account of sampling strategy |  | N/A |
|  |  | (*e*) Describe any sensitivity analyses | 13 | We conducted additional sensitivity analyses to explore whether there was an effect on our results due to the inclusion of those who previously drank alcohol but who may have quit due to health issues (“sick quitting”), by excluding participants who had not consumed alcohol in the prior year, but who had reported any past history of alcohol use. Finally, we conducted additional sensitivity analyses to assess whether results may be different by ART type; we conducted multivariable models, adjusted for ART regimen type (NNRTI-based, INSTI-base, PI-based). |
| Results | | | | |
| Participants | 13* | (a) Report numbers of individuals at each stage of study—eg numbers potentially eligible, examined for eligibility, confirmed eligible, included in the study, completing follow-up, and analysed | 14 | The DIPT (n=680) and ADEPTT (n=301) studies enrolled participants from May 2017 through August 2021. Seventeen of the 680 persons enrolled in the DIPT study did not have viral load results leaving 663 for analysis; nine of 301 participants from the ADEPTT study were missing viral load results leaving 292 for analysis, for a total of 955. |
|  |  | (b) Give reasons for non-participation at each stage | 14 | Seventeen of the 680 persons enrolled in the DIPT study did not have viral load results. Nine of 301 participants from the ADEPTT study were missing viral load results leaving 292 for analysis, for a total of 955. |
|  |  | (c) Consider use of a flow diagram |  |  |
| Descriptive data | 14* | 1. Give characteristics of study participants (eg demographic, clinical, social) and information on exposures and potential confounders | 14-17 | The median age was 40 years (interquartile range [IQR]: 32-47 The majority of participants were men (63.4%), married (61.0%), had at most a primary level of education (77.7%), and reported very good to excellent general health (51.4%).  Twenty-two percent (22.2%) reported smoking tobacco in the prior 3 months. For alcohol use, 17.0% were in the abstaining/low risk drinking group, 20.4% were in the medium risk group, and 62.6% were in the high/very high-risk group. Eighty-eight participants (9.2%) had lack of virological suppression and the median log10 viral load was 2.3 copies/ml^3^ (IQR: 1.8-3.7). Among participants with viral suppression (n=867) whose CD4 counts were available (n=865), 10.6% had a CD4 cell count <350 cells/ml^3^ and the median CD4 count in this group was 283 (IQR: 225-324). |
|  |  | 1. Indicate number of participants with missing data for each variable of interest | 14 | Seventeen of the 680 persons enrolled in the DIPT study did not have viral load results leaving 663 for analysis; nine of 301 participants from the ADEPTT study were missing viral load results leaving 292 for analysis, for a total of 955 |
|  |  | (c) *Cohort study*—Summarise follow-up time (eg, average and total amount) |  | N/A |
| Outcome data | 15* | *Cohort study*—Report numbers of outcome events or summary measures over time |  | N/A |
|  |  | *Case-control study—*Report numbers in each exposure category, or summary measures of exposure |  | N/A |
|  |  | *Cross-sectional study—*Report numbers of outcome events or summary measures | 14-17,19-24 | All numbers are reported in Tables 1,2, 3,4&5 |
| Main results | 16 | (*a*) Give unadjusted estimates and, if applicable, confounder-adjusted estimates and their precision (eg, 95% confidence interval). Make clear which confounders were adjusted for and why they were included | 18-19 | In unadjusted analyses, there was no evidence of an association between current smoking or level of alcohol use with viral non-suppression. In the multivariable model examining lack of virological suppression, the adjusted odds ratio (aOR) for lack of virological suppression for persons who currently smoke compared to those who do not currently smoke was 0.74 (95% confidence interval (CI): 0.42-1.32).  In unadjusted analyses among the 865 participants with viral suppression, there was no evidence of an association between current smoking and low CD4 at baseline (OR 0.89; 95% CI: 0.52-1.51). The adjusted aOR for low CD4 for persons who currently smoke was 0.64 (95% CI: 0.37-1.11), compared to those who do not currently smoke. |
|  |  | (*b*) Report category boundaries when continuous variables were categorized |  | N/A |
|  |  | (*c*) If relevant, consider translating estimates of relative risk into absolute risk for a meaningful time period |  | N/A |

Continued on next page

| Other analyses | 17 | Report other analyses done—eg analyses of subgroups and interactions, and sensitivity analyses | 25 | We conducted sensitivity analyses by running multivariable models for lack of virological suppression 1) excluding 54 persons with past alcohol use, and 2) additionally adjusted for ART type. The results were similar to our other findings, i.e., there was no evidence of an independent association of current smoking or level of alcohol use with lack of virological suppression. In similar analyses of low CD4, there was no evidence of an independent association of current smoking or level of alcohol use with low CD4 among those with viral suppression, after excluding those with past alcohol use or adjusting for ART regimen type. |
| --- | --- | --- | --- | --- |
| Discussion | | | | |
| Key results | 18 | Summarise key results with reference to study objectives. | 25-26 | There was a high proportion engaging in smoking (22.2%), which is higher than reported in the general population in Uganda (7.9%). Smoking was more common among men compared to women and among those with high-risk alcohol use. We found no association between smoking and lack of virological suppression; however, viral suppression was high (90.8%).  The proportion with low CD4 count was also low in this population (10.6% had CD4 count<350 copies/mm^3^ among those with viral suppression), and we observed no association between smoking and low CD4 count among those with viral suppression.  We did not find significant associations between smoking and either lack of virological suppression or low CD4, unadjusted or adjusted for alcohol use. |
| Limitations | 19 | Discuss limitations of the study, taking into account sources of potential bias or imprecision. Discuss both direction and magnitude of any potential bias | 26 | This study has several limitations. First, information on smoking was self-reported, and smoking may be under-reported due to social desirability bias and recall bias |
| Interpretation | 20 | Give a cautious overall interpretation of results considering objectives, limitations, multiplicity of analyses, results from similar studies, and other relevant evidence | 26-27 | While smoking was common, especially among the men, we did not find significant associations between smoking and either lack of virological suppression or low CD4, unadjusted or adjusted for alcohol use. This analysis reveals a substantial incidence of tobacco smoking among PLWH who consume alcohol in Southwest Uganda, which constitutes a significant health risk. However, the study indicates that tobacco smoking might not have a direct correlation with viral suppression or CD4 count in PLWH. |
| Generalisability | 21 | Discuss the generalisability (external validity) of the study results | 26 | An explanation for the lack of consistent findings may be that the level of non-adherence needed to result in lack of HIV virological suppression may be quite high, especially with the fairly recent advent and broad uptake of more forgiving second generation integrase strand transfer inhibitor (INSTI)-based regimens. Mixed findings may be partly due to the use of different smoking measures. |
| Other information | |  | | |
| Funding | 22 | Give the source of funding and the role of the funders for the present study and, if applicable, for the original study on which the present article is based | 2 | The study was supported by Grants from the US National Institute of Health/ National Institute of Alcohol Abuse and Alcoholism: Grant U01 AA020776 (Hahn) and U01AA026223 (Hahn) and U01AA026221 (Chamie) and K24AA022586 (Hahn), the Boston/Providence Center for AIDS Research (CFAR) (P30AI042853) |

*Give information separately for cases and controls in case-control studies and, if applicable, for exposed and unexposed groups in cohort and cross-sectional studies.

**Note:** An Explanation and Elaboration article discusses each checklist item and gives methodological background and published examples of transparent reporting. The STROBE checklist is best used in conjunction with this article (freely available on the Web sites of PLoS Medicine at http://www.plosmedicine.org/, Annals of Internal Medicine at http://www.annals.org/, and Epidemiology at http://www.epidem.com/). Information on the STROBE Initiative is available at www.strobe-statement.org.
