# Supplementary material for: Association between smoking and lack of HIV virological suppression in a cross-sectional study of persons with HIV on antiretroviral therapy in Uganda
Source: PLoS One. 2024 Mar 20;19(3):e0300508. doi: 10.1371/journal.pone.0300508 (PMC10954112; doi:10.1371/journal.pone.0300508)
Supplement: Attachment — Submitted filename: Response to Reviewers.docx [file pone.0300508.s003.docx]

**Response to Reviewers, Manuscript # PONE-D-23-14850**

**Title: Association between smoking and lack of HIV virological suppression in a cross-sectional study of persons with HIV who engage in alcohol use on antiretroviral therapy in Uganda.**

**Reviewer #1:**

1. The introduction has very little mention about smoking and more about alcohol. This can be improved.

*Response: The introduction paragraphs 1-3 focus solely on smoking. Paragraph 4 brings in the potentially confounding factor of alcohol on the association of smoking with viral suppression. We have edited paragraph 5 to focus it on the association between smoking and immune status. The paragraph now reads:*

*“Even among those with viral suppression, smoking may contribute to disrupting immune pathways in PLWH [1, 2]. However, the literature regarding the association of smoking with CD4 cell count among PLWH on ART has mixed findings. Some studies have linked smoking with poorer CD4 cell count recovery over time after ART initiation [3-5]. Other literature found no association [6]. Therefore, there is a need to better understand whether smoking impacts CD4 cell count in PLWH who receive ART.”*

Line 97 PSYCHOSOCIAL FACTORS (Depression and Anxiety). I believe a better word(S) will do to describe. Depression and anxiety because these are disorders. Psychosocial factors are way broader than just mental disorders.

*Response: We have changed the text to say, “mental health conditions (depression and anxiety)”.*

Line 99. Should read SMOKERS not SMOKING.

*Response: We agree with the comment and have changed it to read “smokers”*

2. No sample size calculation was included in the manuscript. This ought to be included.

*Response: We did not conduct an ad-hoc power calculation for this study since we didn’t consider power when we planned the study, but combining ADEPTT and DIPT studies allowed us to address our research question in a large sample of patients with HIV, on ART, with latent tuberculosis, and generally high levels of alcohol drinking in Uganda. The protocols of the parent studies and their sample size determination have been published elsewhere[7-9].*

3. Missing data handling besides that mentioned for viral loads was barely mentioned.

*Response: We have added a text to the results section (pg19, line 318-323). “948 participants were included in the multivariable model examining lack of virological suppression. Seven of 955 participants were excluded due to missing data; 6 were missing alcohol use and 1 was missing social desirability score.”*

4. Despite declaration of provision of data without restriction, not all data reported in results was actually available within manuscript and no link to data repository where it could be found was added.

*Response: All data used in this manuscript have now been included with this submission, to be included with the manuscript as “Supporting Information” files.*

**Reviewer #2:**

1. Please include your study aims at the end of the introduction in the abstract section.

*Response: We now included the aims at the end of the introduction in the abstract section as follows: “We aimed to 1) describe the prevalence of current smoking and correlates of smoking; 2) assess the association of smoking with viral suppression, adjusting for level of alcohol use; 3) explore the relationship between smoking and CD4 cell count <350 cells/mm3, among participants who are virally suppressed”*

2. I think the phrase "who engage in alcohol use" in the title is quite misleading since not all your participants engage in alcohol use.

*Response: We have changed it to read “Association between smoking and lack of HIV virological suppression in a cross-sectional study of persons with HIV on antiretroviral therapy in Uganda”*

3. Your main study outcome (cigarette use) is assessed by self-report, and you acknowledge this in your limitations. Are there no tools that assess for cigarette use.

*Response: There are tools that assess for cigarette use but unfortunately this was not the focus of the studies.*

*We have added a text to the Discussion (limitations section) regarding not using a biomarker for smoking as follows (page 27, line 416-418).*

*“We did not include a biomarker for smoking to augment self-report, since it was not the focus of these studies.”*

4. The English grammar in the introduction section needs to be improved.

*Response: Thanks, we have made edits to the introduction section to improve grammatical clarity.*

5. In lines 152-152, you indicate that the study participants were followed up, yet in the title you noted that this was a cross-section study.

*Response: This analysis used only baseline data from the parent studies. Thus, we have changed the text to read (page 8, line 158-159): “We recruited participants from May 2017 through August 2021.”*

6. Lines 210-212. The statements here do not make sense.

*Response: We have rephrased to read “Persons with past alcohol use were defined as participants who reported no past year alcohol use (AUDIT =0) and who had any past history of alcohol use”.*

7. In table 1, you only reported primary and secondary level of education. Where there no participants with tertiary level education.

*Response: We dichotomized level of education as "any primary school", versus "secondary school or more". Forty participants reported higher than secondary school education and were included in the "secondary school or more" group. We have re-phrased these groups in the manuscript to be “primary school only” and “secondary school or higher”. We have also now excluded the level of education and a few other variables from Table 1, in response to a later comment.*

8. The reference to the term "baseline" especially in the results is confusing since this was a cross sectional study.

*Response: We agree with the comment, and we have deleted the term “baseline” from the results section.*

9. The tables are generally too long spanning 2-4 pages. please edit them accordingly and improve their quality.

*Response: We have shortened the tables and improved their quality.*

10. It may be useful to define the term current "cigarette use" because you seem to measure days of cigarette use and use them interchangeably with current cigarette use.

*Response: We have clarified this definition in the Methods Section on page 10, as follows: “We ascertained current smoking status during the structured interview by asking participants whether they had smoked in the past 3 months (yes/no). Participants reporting current smoking were also asked to report the number of smoking days in the prior 30 days.”*

**Reviewer #3**

1. One of the variables assessed was the general physical health of the study participants. How was this measured? It would be good to provide this information under the variables section of the paper, just as was done for other variables.

*Response: We have added to the variable section as follows (page 12, line 233-236): We assessed general health status using the first question of the Medical Outcomes Study-HIV (MOS-HIV) Health Survey that asked; In general, would you say your health is excellent, very good, good, fair, or poor? The MOS-HIV has been used before as a validated measure of health in Uganda.*

2. Regarding statistical analysis, why did the authors only conduct bivariable analysis when looking for correlates of smoking (objective 1)? Why didn’t they conduct any multivariable analysis?

*Response: The primary objective was to examine the association between smoking and HIV viral suppression and CD4. We examined correlates of smoking descriptively.*

3. In the Study Design and setting section, the authors talk about analyzing data from “…two cross-sectional studies of PLWH….” However, the two studies that they go on to describe are trials. This may be confusing to the reader. Can you provide clarification on this?

*Response: To make this more clear, we now state (page 7, line 141-142) “We used baseline data from two longitudinal studies”*

***References cited in the responses above:***

1.	Hasson H, Saniabadi A, Alfano M, Trabattoni D, Ferrante P, Lillo F, et al. Granulocyte/monocyte apheresis induces sustained increases in CD4 T cells in HIV-1 infected patients with poor CD4 T cell restoration after suppression of viral replication by HAART. Journal of Biological Regulators and Homeostatic Agents. 2002;16(1):58-63.

2.	Monnig MA. Immune activation and neuroinflammation in alcohol use and HIV infection: evidence for shared mechanisms. The American journal of drug and alcohol abuse. 2017;43(1):7-23.

3.	Estevez A, Taveras Soriano GA, Rodríguez Velazquez A, Díaz S, Quiñones de Monegro Z, Sánchez JJ. Relationship of Smoking with the CD4+ T cell count and Viral Load in Patients with the Human Immunodeficiency Virus in the HIV health care center at the Hospital Regional Universitario José María Cabral y Báez in the Dominican Republic: a Cross-sectional Descriptive Study. Infectio. 2021;25(2):79-83.

4.	Feldman JG, Minkoff H, Schneider MF, Gange SJ, Cohen M, Watts DH, et al. Association of cigarette smoking with HIV prognosis among women in the HAART era: a report from the women's interagency HIV study. Am J Public Health. 2006;96(6):1060-5.

5.	Winhusen T, Feaster DJ, Duan R, Brown JL, Daar ES, Mandler R, et al. Baseline Cigarette Smoking Status as a Predictor of Virologic Suppression and CD4 Cell Count During One-Year Follow-Up in Substance Users with Uncontrolled HIV Infection. AIDS Behav. 2018;22(6):2026-32.

6.	Brown JL, Winhusen T, DiClemente RJ, Sales JM, Rose ES, Safonova P, et al. The association between cigarette smoking, virologic suppression, and CD4+ lymphocyte count in HIV-Infected Russian women. AIDS Care. 2017;29(9):1102-6.

7.	Chamie G, Hahn JA, Kekibiina A, Emenyonu NI, Beesiga B, Marson K, et al. Financial incentives for reduced alcohol use and increased isoniazid adherence during tuberculosis preventive therapy among people with HIV in Uganda: an open-label, factorial randomised controlled trial. Lancet Glob Health. 2023;11(12):e1899-e910.

8.	Hahn JA, Ngabirano C, Fatch R, Emenyonu NI, Cheng DM, Adong J, et al. Safety and tolerability of isoniazid preventive therapy for tuberculosis for persons with HIV with and without alcohol use. Aids. 2023;37(10):1535-43.

9.	Lodi S, Emenyonu NI, Marson K, Kwarisiima D, Fatch R, McDonell MG, et al. The Drinkers' Intervention to Prevent Tuberculosis (DIPT) trial among heavy drinkers living with HIV in Uganda: study protocol of a 2x2 factorial trial. Trials. 2021;22(1):355.
