# Supplementary material for: Association between smoking and lack of HIV virological suppression in a cross-sectional study of persons with HIV on antiretroviral therapy in Uganda
Source: PLoS One. 2024 Mar 20;19(3):e0300508. doi: 10.1371/journal.pone.0300508 (PMC10954112; doi:10.1371/journal.pone.0300508)
Supplement: Peer review history

# Peer review: Decision Letter 0

**DOI:** 10.1371/journal.pone.0300508.r001

**Authors:** Dickens Akena

28 Nov 2023

PONE-D-23-14850Association between smoking and lack of HIV virological suppression in a cross-sectional study of persons with HIV who engage in alcohol use on antiretroviral therapy in Uganda.PLOS ONE

Dear Dr. Hahn,

Thank you for submitting your manuscript to PLOS ONE. After careful consideration, we feel that it has merit but does not fully meet PLOS ONE’s publication criteria as it currently stands. Therefore, we invite you to submit a revised version of the manuscript that addresses the points raised during the review process.

Please submit your revised manuscript by Jan 12 2024 11:59PM. If you will need more time than this to complete your revisions, please reply to this message or contact the journal office at plosone@plos.org. When you're ready to submit your revision, log on to https://www.editorialmanager.com/pone/ and select the 'Submissions Needing Revision' folder to locate your manuscript file.

Please include the following items when submitting your revised manuscript:

- A rebuttal letter that responds to each point raised by the academic editor and reviewer(s). You should upload this letter as a separate file labeled 'Response to Reviewers'.
- A marked-up copy of your manuscript that highlights changes made to the original version. You should upload this as a separate file labeled 'Revised Manuscript with Track Changes'.
- An unmarked version of your revised paper without tracked changes. You should upload this as a separate file labeled 'Manuscript'.

If you would like to make changes to your financial disclosure, please include your updated statement in your cover letter. Guidelines for resubmitting your figure files are available below the reviewer comments at the end of this letter.

If applicable, we recommend that you deposit your laboratory protocols in protocols.io to enhance the reproducibility of your results. Protocols.io assigns your protocol its own identifier (DOI) so that it can be cited independently in the future. For instructions see: https://journals.plos.org/plosone/s/submission-guidelines#loc-laboratory-protocols. Additionally, PLOS ONE offers an option for publishing peer-reviewed Lab Protocol articles, which describe protocols hosted on protocols.io. Read more information on sharing protocols at https://plos.org/protocols?utm_medium=editorial-email&utm_source=authorletters&utm_campaign=protocols.

We look forward to receiving your revised manuscript.

Kind regards,

Dickens Akena, Ph.D

Academic Editor

PLOS ONE

Journal Requirements:

When submitting your revision, we need you to address these additional requirements.

1. Please ensure that your manuscript meets PLOS ONE's style requirements, including those for file naming. The PLOS ONE style templates can be found at

https://journals.plos.org/plosone/s/file?id=wjVg/PLOSOne_formatting_sample_main_body.pdf and

https://journals.plos.org/plosone/s/file?id=ba62/PLOSOne_formatting_sample_title_authors_affiliations.pdf

2. Note from Emily Chenette, Editor in Chief of PLOS ONE, and Iain Hrynaszkiewicz, Director of Open Research Solutions at PLOS: Did you know that depositing data in a repository is associated with up to a 25% citation advantage (https://doi.org/10.1371/journal.pone.0230416)? If you’ve not already done so, consider depositing your raw data in a repository to ensure your work is read, appreciated and cited by the largest possible audience. You’ll also earn an Accessible Data icon on your published paper if you deposit your data in any participating repository (https://plos.org/open-science/open-data/#accessible-data).

3. We suggest you thoroughly copyedit your manuscript for language usage, spelling, and grammar. If you do not know anyone who can help you do this, you may wish to consider employing a professional scientific editing service. 

Whilst you may use any professional scientific editing service of your choice, PLOS has partnered with both American Journal Experts (AJE) and Editage to provide discounted services to PLOS authors. Both organizations have experience helping authors meet PLOS guidelines and can provide language editing, translation, manuscript formatting, and figure formatting to ensure your manuscript meets our submission guidelines. To take advantage of our partnership with AJE, visit the AJE website (http://learn.aje.com/plos/) for a 15% discount off AJE services. To take advantage of our partnership with Editage, visit the Editage website (www.editage.com) and enter referral code PLOSEDIT for a 15% discount off Editage services.  If the PLOS editorial team finds any language issues in text that either AJE or Editage has edited, the service provider will re-edit the text for free.

Upon resubmission, please provide the following:

● The name of the colleague or the details of the professional service that edited your manuscript

● A copy of your manuscript showing your changes by either highlighting them or using track changes (uploaded as a *supporting information* file)

● A clean copy of the edited manuscript (uploaded as the new *manuscript* file)

4. We note that the grant information you provided in the ‘Funding Information’ and ‘Financial Disclosure’ sections do not match.

When you resubmit, please ensure that you provide the correct grant numbers for the awards you received for your study in the ‘Funding Information’ section.

5. Please include your full ethics statement in the ‘Methods’ section of your manuscript file. In your statement, please include the full name of the IRB or ethics committee who approved or waived your study, as well as whether or not you obtained informed written or verbal consent. If consent was waived for your study, please include this information in your statement as well.

6. We note that you have stated that you will provide repository information for your data at acceptance. Should your manuscript be accepted for publication, we will hold it until you provide the relevant accession numbers or DOIs necessary to access your data. If you wish to make changes to your Data Availability statement, please describe these changes in your cover letter and we will update your Data Availability statement to reflect the information you provide.

7. Please include captions for your Supporting Information files at the end of your manuscript, and update any in-text citations to match accordingly. Please see our Supporting Information guidelines for more information: http://journals.plos.org/plosone/s/supporting-information.

8. Please review your reference list to ensure that it is complete and correct. If you have cited papers that have been retracted, please include the rationale for doing so in the manuscript text, or remove these references and replace them with relevant current references. Any changes to the reference list should be mentioned in the rebuttal letter that accompanies your revised manuscript. If you need to cite a retracted article, indicate the article’s retracted status in the References list and also include a citation and full reference for the retraction notice.

Additional Editor Comments:

The reviewers have returned their reports and recommend a minor correction. Please ensure that you respond to the comments.

Kind regards

[Note: HTML markup is below. Please do not edit.]

Reviewers' comments:

Reviewer's Responses to Questions

**Comments to the Author**

1. Is the manuscript technically sound, and do the data support the conclusions?

The manuscript must describe a technically sound piece of scientific research with data that supports the conclusions. Experiments must have been conducted rigorously, with appropriate controls, replication, and sample sizes. The conclusions must be drawn appropriately based on the data presented. 

Reviewer #1: Yes

Reviewer #2: Yes

Reviewer #3: Yes

**********

2. Has the statistical analysis been performed appropriately and rigorously? 

Reviewer #1: Yes

Reviewer #2: I Don't Know

Reviewer #3: Yes

**********

3. Have the authors made all data underlying the findings in their manuscript fully available?

The PLOS Data policy requires authors to make all data underlying the findings described in their manuscript fully available without restriction, with rare exception (please refer to the Data Availability Statement in the manuscript PDF file). The data should be provided as part of the manuscript or its supporting information, or deposited to a public repository. For example, in addition to summary statistics, the data points behind means, medians and variance measures should be available. If there are restrictions on publicly sharing data—e.g. participant privacy or use of data from a third party—those must be specified.

Reviewer #1: No

Reviewer #2: Yes

Reviewer #3: Yes

**********

4. Is the manuscript presented in an intelligible fashion and written in standard English?

PLOS ONE does not copyedit accepted manuscripts, so the language in submitted articles must be clear, correct, and unambiguous. Any typographical or grammatical errors should be corrected at revision, so please note any specific errors here.

Reviewer #1: Yes

Reviewer #2: Yes

Reviewer #3: Yes

**********

5. Review Comments to the Author

Please use the space provided to explain your answers to the questions above. You may also include additional comments for the author, including concerns about dual publication, research ethics, or publication ethics. (Please upload your review as an attachment if it exceeds 20,000 characters)

Reviewer #1: Generally a good paper with rigorous analysis that adds the body of knowledge. A fairly coherent thought process is shown from the justification for the study, objectives, choice of methods, analyses and results.

I however feel the following could have been done better.

1. The introduction has very little mention about smoking and more about alcohol. This can be improved.

Line 97 PSYCHOSOCIAL FACTORS (Depression and Anxiety). I believe a better word(S) will do to describe

Depression and anxiety because these are disorders. Psychosocial factors are way broader than just mental

disorders.

Line 99. Should read SMOKERS not SMOKING.

2. No sample size calculation was included in the manuscript. This ought to be included.

3. Missing data handling besides that mentioned for Viral loads was barely mentioned.

4. Despite declaration of provision of data without restriction, not all data reported in results was actually available

within manuscript and no link to data repository where it could be found was added.

Reviewer #2: Thank you for this important research. here are more comments

1. please include your study aims at the end of the introduction in the abstract section.

2. I think the phrase "who engage in alcohol use" in the title is quite misleading since not all your participants engage in alcohol use

3. Your main study outcome (cigarette use) is assessed by self report and you acknowledge this in your limitations. Are there no tools that assess for cigarette use

4. The English grammar in the introduction section needs to be improved

5. in lines 152-152, you indicate that the study participants were followed up, yet in the title you noted that this was a cross-section study.

6. lines 210-212. The statements here do not make sense

7. in table 1, you only reported primary and secondary level of education. Where there no participants with tertiary level education

8. the reference to the term "baseline" especially in the results is confusing since this was a cross sectional study

9. The tables are generally too long spanning 2-4 pages. please edit them accordingly and improve their quality.

10. it may be useful to define the term current "cigarette use" because you seem to measure days of cigarette use and use them interchangeably with current cigarette use.

Reviewer #3: This is an interesting and well written paper. I have a few comments/ questions.

1. One of the variables assessed was the general physical health of the study participants. How was this measured? It would be good to provide this information under the variables section of the paper, just as was done for other variables.

2. Regarding statistical analysis, why did the authors only conduct bivariable analysis when looking for correlates of smoking (objective 1)? Why didn’t they conduct any multivariable analysis?

3. In the Study Design and setting section, the authors talk about analyzing data from “…two cross-sectional studies of PLWH….” However, the two studies that they go on to describe are trials. This may be confusing to the reader. Can you provide clarification on this?

**********

6. PLOS authors have the option to publish the peer review history of their article (what does this mean?). If published, this will include your full peer review and any attached files.

If you choose “no”, your identity will remain anonymous but your review may still be made public.

**Do you want your identity to be public for this peer review?** For information about this choice, including consent withdrawal, please see our Privacy Policy.

Reviewer #1: No

Reviewer #2: No

Reviewer #3: No

**********

[NOTE: If reviewer comments were submitted as an attachment file, they will be attached to this email and accessible via the submission site. Please log into your account, locate the manuscript record, and check for the action link "View Attachments". If this link does not appear, there are no attachment files.]

While revising your submission, please upload your figure files to the Preflight Analysis and Conversion Engine (PACE) digital diagnostic tool, https://pacev2.apexcovantage.com/. PACE helps ensure that figures meet PLOS requirements. To use PACE, you must first register as a user. Registration is free. Then, login and navigate to the UPLOAD tab, where you will find detailed instructions on how to use the tool. If you encounter any issues or have any questions when using PACE, please email PLOS at figures@plos.org. Please note that Supporting Information files do not need this step.

# Author comment: Author response to Decision Letter 0

**DOI:** 10.1371/journal.pone.0300508.r002

14 Feb 2024

Reviewer #1: 

1. The introduction has very little mention about smoking and more about alcohol. This can be improved.

Response: The introduction paragraphs 1-3 focus solely on smoking. Paragraph 4 brings in the potentially confounding factor of alcohol on the association of smoking with viral suppression. We have edited paragraph 5 to focus it on the association between smoking and immune status. The paragraph now reads:

“Even among those with viral suppression, smoking may contribute to disrupting immune pathways in PLWH [1, 2]. However, the literature regarding the association of smoking with CD4 cell count among PLWH on ART has mixed findings. Some studies have linked smoking with poorer CD4 cell count recovery over time after ART initiation [3-5]. Other literature found no association [6]. Therefore, there is a need to better understand whether smoking impacts CD4 cell count in PLWH who receive ART.”

Line 97 PSYCHOSOCIAL FACTORS (Depression and Anxiety). I believe a better word(S) will do to describe. Depression and anxiety because these are disorders. Psychosocial factors are way broader than just mental disorders.

Response: We have changed the text to say, “mental health conditions (depression and anxiety)”.

Line 99. Should read SMOKERS not SMOKING.

Response: We agree with the comment and have changed it to read “smokers”

2. No sample size calculation was included in the manuscript. This ought to be included.

Response: We did not conduct an ad-hoc power calculation for this study since we didn’t consider power when we planned the study, but combining ADEPTT and DIPT studies allowed us to address our research question in a large sample of patients with HIV, on ART, with latent tuberculosis, and generally high levels of alcohol drinking in Uganda. The protocols of the parent studies and their sample size determination have been published elsewhere[7-9].

3. Missing data handling besides that mentioned for viral loads was barely mentioned.

Response: We have added a text to the results section (pg19, line 318-323). “948 participants were included in the multivariable model examining lack of virological suppression. Seven of 955 participants were excluded due to missing data; 6 were missing alcohol use and 1 was missing social desirability score.”

4. Despite declaration of provision of data without restriction, not all data reported in results was actually available within manuscript and no link to data repository where it could be found was added.

Response: All data used in this manuscript have now been included with this submission, to be included with the manuscript as “Supporting Information” files.

Reviewer #2: 

1. Please include your study aims at the end of the introduction in the abstract section.

Response: We now included the aims at the end of the introduction in the abstract section as follows: “We aimed to 1) describe the prevalence of current smoking and correlates of smoking; 2) assess the association of smoking with viral suppression, adjusting for level of alcohol use; 3) explore the relationship between smoking and CD4 cell count <350 cells/mm3, among participants who are virally suppressed”

2. I think the phrase "who engage in alcohol use" in the title is quite misleading since not all your participants engage in alcohol use.

Response: We have changed it to read “Association between smoking and lack of HIV virological suppression in a cross-sectional study of persons with HIV on antiretroviral therapy in Uganda”

3. Your main study outcome (cigarette use) is assessed by self-report, and you acknowledge this in your limitations. Are there no tools that assess for cigarette use.

Response: There are tools that assess for cigarette use but unfortunately this was not the focus of the studies. 

We have added a text to the Discussion (limitations section) regarding not using a biomarker for smoking as follows (page 27, line 416-418). 

“We did not include a biomarker for smoking to augment self-report, since it was not the focus of these studies.”

4. The English grammar in the introduction section needs to be improved.

Response: Thanks, we have made edits to the introduction section to improve grammatical clarity.

5. In lines 152-152, you indicate that the study participants were followed up, yet in the title you noted that this was a cross-section study.

Response: This analysis used only baseline data from the parent studies. Thus, we have changed the text to read (page 8, line 158-159): “We recruited participants from May 2017 through August 2021.”

6. Lines 210-212. The statements here do not make sense.

Response: We have rephrased to read “Persons with past alcohol use were defined as participants who reported no past year alcohol use (AUDIT =0) and who had any past history of alcohol use”.

7. In table 1, you only reported primary and secondary level of education. Where there no participants with tertiary level education.

Response: We dichotomized level of education as "any primary school", versus "secondary school or more". Forty participants reported higher than secondary school education and were included in the "secondary school or more" group. We have re-phrased these groups in the manuscript to be “primary school only” and “secondary school or higher”. We have also now excluded the level of education and a few other variables from Table 1, in response to a later comment.

8. The reference to the term "baseline" especially in the results is confusing since this was a cross sectional study.

Response: We agree with the comment, and we have deleted the term “baseline” from the results section.

9. The tables are generally too long spanning 2-4 pages. please edit them accordingly and improve their quality.

Response: We have shortened the tables and improved their quality.

10. It may be useful to define the term current "cigarette use" because you seem to measure days of cigarette use and use them interchangeably with current cigarette use.

Response: We have clarified this definition in the Methods Section on page 10, as follows: “We ascertained current smoking status during the structured interview by asking participants whether they had smoked in the past 3 months (yes/no). Participants reporting current smoking were also asked to report the number of smoking days in the prior 30 days.”

Reviewer #3 

1. One of the variables assessed was the general physical health of the study participants. How was this measured? It would be good to provide this information under the variables section of the paper, just as was done for other variables.

Response: We have added to the variable section as follows (page 12, line 233-236): We assessed general health status using the first question of the Medical Outcomes Study-HIV (MOS-HIV) Health Survey that asked; In general, would you say your health is excellent, very good, good, fair, or poor? The MOS-HIV has been used before as a validated measure of health in Uganda.

2. Regarding statistical analysis, why did the authors only conduct bivariable analysis when looking for correlates of smoking (objective 1)? Why didn’t they conduct any multivariable analysis?

Response: The primary objective was to examine the association between smoking and HIV viral suppression and CD4. We examined correlates of smoking descriptively.

3. In the Study Design and setting section, the authors talk about analyzing data from “…two cross-sectional studies of PLWH….” However, the two studies that they go on to describe are trials. This may be confusing to the reader. Can you provide clarification on this?

Response: To make this more clear, we now state (page 7, line 141-142) “We used baseline data from two longitudinal studies”

References cited in the responses above:

1. Hasson H, Saniabadi A, Alfano M, Trabattoni D, Ferrante P, Lillo F, et al. Granulocyte/monocyte apheresis induces sustained increases in CD4 T cells in HIV-1 infected patients with poor CD4 T cell restoration after suppression of viral replication by HAART. Journal of Biological Regulators and Homeostatic Agents. 2002;16(1):58-63.

2. Monnig MA. Immune activation and neuroinflammation in alcohol use and HIV infection: evidence for shared mechanisms. The American journal of drug and alcohol abuse. 2017;43(1):7-23.

3. Estevez A, Taveras Soriano GA, Rodríguez Velazquez A, Díaz S, Quiñones de Monegro Z, Sánchez JJ. Relationship of Smoking with the CD4+ T cell count and Viral Load in Patients with the Human Immunodeficiency Virus in the HIV health care center at the Hospital Regional Universitario José María Cabral y Báez in the Dominican Republic: a Cross-sectional Descriptive Study. Infectio. 2021;25(2):79-83.

4. Feldman JG, Minkoff H, Schneider MF, Gange SJ, Cohen M, Watts DH, et al. Association of cigarette smoking with HIV prognosis among women in the HAART era: a report from the women's interagency HIV study. Am J Public Health. 2006;96(6):1060-5.

5. Winhusen T, Feaster DJ, Duan R, Brown JL, Daar ES, Mandler R, et al. Baseline Cigarette Smoking Status as a Predictor of Virologic Suppression and CD4 Cell Count During One-Year Follow-Up in Substance Users with Uncontrolled HIV Infection. AIDS Behav. 2018;22(6):2026-32.

6. Brown JL, Winhusen T, DiClemente RJ, Sales JM, Rose ES, Safonova P, et al. The association between cigarette smoking, virologic suppression, and CD4+ lymphocyte count in HIV-Infected Russian women. AIDS Care. 2017;29(9):1102-6.

7. Chamie G, Hahn JA, Kekibiina A, Emenyonu NI, Beesiga B, Marson K, et al. Financial incentives for reduced alcohol use and increased isoniazid adherence during tuberculosis preventive therapy among people with HIV in Uganda: an open-label, factorial randomised controlled trial. Lancet Glob Health. 2023;11(12):e1899-e910.

8. Hahn JA, Ngabirano C, Fatch R, Emenyonu NI, Cheng DM, Adong J, et al. Safety and tolerability of isoniazid preventive therapy for tuberculosis for persons with HIV with and without alcohol use. Aids. 2023;37(10):1535-43.

9. Lodi S, Emenyonu NI, Marson K, Kwarisiima D, Fatch R, McDonell MG, et al. The Drinkers' Intervention to Prevent Tuberculosis (DIPT) trial among heavy drinkers living with HIV in Uganda: study protocol of a 2x2 factorial trial. Trials. 2021;22(1):355.

Attachment Submitted filename: Response to Reviewers.docx

# Editor report: Decision Letter 1

**DOI:** 10.1371/journal.pone.0300508.r003

**Authors:** Dickens Akena

29 Feb 2024

Association between smoking and lack of HIV virological suppression in a cross-sectional study of persons with HIV on antiretroviral therapy in Uganda.

PONE-D-23-14850R1

Dear Dr. Hahn,

We’re pleased to inform you that your manuscript has been judged scientifically suitable for publication and will be formally accepted for publication once it meets all outstanding technical requirements.

Within one week, you’ll receive an e-mail detailing the required amendments. When these have been addressed, you’ll receive a formal acceptance letter and your manuscript will be scheduled for publication.

An invoice for payment will follow shortly after the formal acceptance. To ensure an efficient process, please log into Editorial Manager at http://www.editorialmanager.com/pone/, click the 'Update My Information' link at the top of the page, and double check that your user information is up-to-date. If you have any billing related questions, please contact our Author Billing department directly at authorbilling@plos.org.

If your institution or institutions have a press office, please notify them about your upcoming paper to help maximize its impact. If they’ll be preparing press materials, please inform our press team as soon as possible -- no later than 48 hours after receiving the formal acceptance. Your manuscript will remain under strict press embargo until 2 pm Eastern Time on the date of publication. For more information, please contact onepress@plos.org.

Kind regards,

Dickens Akena, Ph.D

Academic Editor

PLOS ONE

Additional Editor Comments (optional):

Reviewers' comments:

# Editor report: Acceptance letter

**DOI:** 10.1371/journal.pone.0300508.r004

**Authors:** Dickens Akena

11 Mar 2024

PONE-D-23-14850R1 

PLOS ONE

Dear Dr. Hahn, 

I'm pleased to inform you that your manuscript has been deemed suitable for publication in PLOS ONE. Congratulations! Your manuscript is now being handed over to our production team.

At this stage, our production department will prepare your paper for publication. This includes ensuring the following:

* All references, tables, and figures are properly cited

* All relevant supporting information is included in the manuscript submission,

* There are no issues that prevent the paper from being properly typeset

If revisions are needed, the production department will contact you directly to resolve them. If no revisions are needed, you will receive an email when the publication date has been set. At this time, we do not offer pre-publication proofs to authors during production of the accepted work. Please keep in mind that we are working through a large volume of accepted articles, so please give us a few weeks to review your paper and let you know the next and final steps. 

Lastly, if your institution or institutions have a press office, please let them know about your upcoming paper now to help maximize its impact. If they'll be preparing press materials, please inform our press team within the next 48 hours. Your manuscript will remain under strict press embargo until 2 pm Eastern Time on the date of publication. For more information, please contact onepress@plos.org.

If we can help with anything else, please email us at customercare@plos.org.

Thank you for submitting your work to PLOS ONE and supporting open access. 

Kind regards, 

PLOS ONE Editorial Office Staff

on behalf of

Dr. Dickens Akena 

Academic Editor

PLOS ONE
